# The Froude-Carlyle Controversy

**Published:** 1903-08

**Authors:** 


### *EDITORIAL NOTES AND COMMENTS.*

The Business office of THE JOURNAL-RECORD is No. 1007-1008 Century Building.

The Editorial office is 318-319-320 The Prudential.

Address all Business communications to Dr. M. B. Hutchins, Mgr.

Make remittances payable to THE ATLANTA JOURNAL-RECORD OF MEDICINE.

On matters pertaining to the Editorial and Original Communications address Dr. Bernard Wolff, Atlanta.

Reprints of original articles will be furnished at cost price. Requests for the same should always be made *on the manuscript.*

We will present, post-paid, on request, to each contributor of an original article, twenty (20) marked copies of THE JOURNAL-RECORD containing such article.

### THE FROUDE-CARLYLE CONTROVERSY.

MANY are doubtless aware that there have been for a long time hints that back of the crabbed ill-temper showing through the rugged English of Carlyle's writings there was a physical, genital defect in part responsible for his dark moods. This hint assumed definite form in the blunt assertion of its actual existence by his biographer, the late James Anthony Froude. Froude based his statement upon hearsay evidence from various sources and more particularly upon the alleged confession of Jane Welsh Carlyle to her friend, Miss Geraldine Jewsbury, an hysterical novelist of the erotic type, that Carlyle had never performed his full duty as a husband.

Froude regards this defect as the cause of Carlyle's misanthropy and the occasion of his practical separation from his wife for years before her death.

On the other hand, Sir James Crichton-Browne (a medical man) in the columns of the *British Medical Journal* scouts at the idea of Carlyle's impotence, and vigorously assails the motive and the veracity of the dead biographer of the dead writer. He calls Froude's assertion a "foul imputation," and uses other expressions equally forcible.

Sir Crichton-Browne is, however, not free from drawing conclusions from sources of hearsay evidence which he so denounces in Froude, and the reader completes the perusal of the argument in an uncertainty of mind as to the preponderance of belief in the accusation or the rebuttal. The facts are apparent from what Carlyle's physician, as well as his truss maker (for he was the subject of hernia), said in regard to physical conformation, that he was normally constructed, and that his wife was sprung from a highly neurotic family and that she was herself exceedingly neurasthenic. The latter condition, united with Carlyle's own nervous instability, were certainly sufficient to lay the foundation for marital infelicity and to establish a breech that widened with the years.

But for what purpose beyond satisfying an idle pruriency is the controversy? Why seek to change the enthusiasm of many for Carlyle to contempt for the Eunuch? Carlyle applied the scourge to himself in life, and why not allow him to remain enshrined in that eternal peace God gives to those who need it most?

